# Three-Factor Fast Authentication Scheme with Time Bound and User Anonymity for Multi-Server E-Health Systems in 5G-Based Wireless Sensor Networks

**DOI:** 10.3390/s20092511

**Published:** 2020-04-29

**Authors:** Alice May-Kuen Wong, Chien-Lung Hsu, Tuan-Vinh Le, Mei-Chen Hsieh, Tzu-Wei Lin

**Affiliations:** 1 Department of Physical Medicine and Rehabilitation, Chang Gung Memorial Hospital, Taoyuan 33302, Taiwan; walice@adm.cgmh.org.tw; 2 Graduate Institute of Business and Management, Chang Gung University, Taoyuan 33302, Taiwan; tvle.cgu@gmail.com (T.-V.L.); d0640001@cgu.edu.tw (T.-W.L.); 3 Department of Information Management, Chang Gung University, Taoyuan 33302, Taiwan; meichen966@gmail.com; 4 Healthy Aging Research Center, Chang Gung University, Taoyuan 33302, Taiwan; 5 Department of Visual Communication Design, Ming Chi University of Technology, New Taipei 24301, Taiwan; 6 Department of Nursing, Taoyuan Chang Gung Memorial Hospital, Taoyuan 33302, Taiwan

**Keywords:** 5G-based WSN, biometrics, multi-server, privacy protection, time bound

## Abstract

The fifth generation (5G) mobile network delivers high peak data rates with ultra-low latency and massive network capacity. Wireless sensor network (WSN) in Internet of Thing (IoT) architecture is of prominent use in 5G-enabled applications. The electronic healthcare (e-health) system has gained a lot of research attention since it allows e-health users to store and share data in a convenient way. By the support of 5G technology, healthcare data produced by sensor nodes are transited in the e-health system with high efficiency and reliability. It helps in reducing the treatment cost, providing efficient services, better analysis reports, and faster access to treatment. However, security and privacy issues become big concerns when the number of sensors and mobile devices is increasing. Moreover, existing single-server architecture requires to store a massive number of identities and passwords, which causes a significant database cost. In this paper, we propose a three-factor fast authentication scheme with time bound and user anonymity for multi-server e-health systems in 5G-based wireless sensor networks. In our work, the three-factor authentication scheme integrating biometrics, password, and smart card ensures a high-security sensor-enabled environment for communicating parties. User anonymity is preserved during communication process. Besides, time bound authentication can be applied to various healthcare scenarios to enhance security. The proposed protocol includes fast authentication, which can provide a fast communication for participating parties. Our protocol is also designed with multi-server architecture to simplify network load and significantly save database cost. Furthermore, security proof and performance analysis results show that our proposed protocol can resist various attacks and bear a rational communication cost.

## 1. Introduction

**Figure 1:**
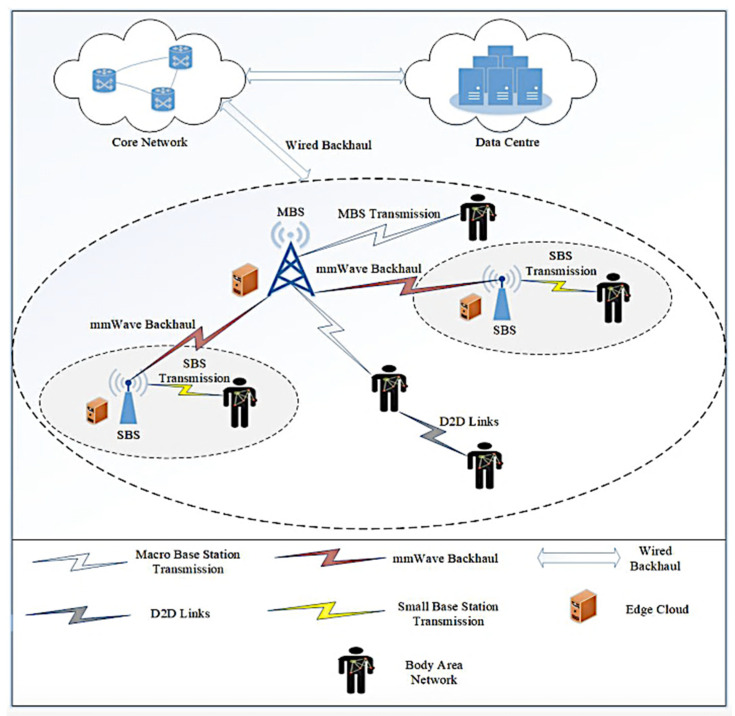
An overview of 5G-based smart healthcare architecture [1].

The fifth generation (5G) mobile network is wireless communication technology supporting two-tier heterogeneous cellular networks (HetNets) with integrated access and backhaul (IAB). As shown in Figure 1, the macro base stations (MBSs) in 5G architecture provide mm-wave backhaul to the small cell base stations (SBSs). Besides, the devices can access both MBSs and SBSs through direction communications [1,2,3]. 5G-enabled devices can also directly communicate with each other. Thus, 5G technology delivers high peak data rates with ultra-low latency and massive network capacity. The Narrow-Band Internet of Things (NB-IoT) system provides low power consumption, wide coverage, low cost, and large capacity, which are essential properties for 5G network [4]. Wireless sensor networks (WSNs) are a key technological building block of IoT, where each object (virtual or physical) can be sensed, identified, accessed, and interconnected via the Internet within a dynamic ubiquitous network [5,6]. WSN applications in distributed IoT architecture can be seen in various domains, such as healthcare [7,8,9], energy [10,11], industrial data acquisition and transmission system [12], mushroom humidity monitoring system [13], intelligent manhole cover monitoring system [14], intelligent station area recognition technology [15], smart car parking system [16], and so on.

The use of IoT in electronic healthcare (e-health) management systems has attracted more and more attention because of its convenience, in which healthcare data are flexibly stored and shared among participating parties. Such a system is called IoMT (Internet of Medical Things) [17,18,19]. IoMT consists of various entities including healthcare centers, emergency centers, medical devices, and e-health users (including patients, physicians, pharmacists, medical researchers, etc.). A Wireless Body Area Network (WBAN) is composed by sensor/actuators nodes and hubs that operates in, on, or around a body (but not limited to human bodies) and supports a variety of medical and non-medical applications [20]. The 5G wireless system aims to support WBAN by increasing the interconnectivity of electronic devices [21].

In e-health systems enabled with 5G-based WSNs, users communicate with servers through a public channel; therefore, their information could be vulnerable to certain attacks, such as man-in-the-middle attack [22], replay attack [23], or impersonation attack [24]. User privacy is also a big issue, where sensitive information of the user may be revealed to the public during communication process. Additionally, existing authentication protocols are not consistent with certain scenarios in healthcare domains, for instance, medical examination appointment, since they were not designed with a time-based mechanism. In addition, most existing authentication protocols were designed with two-factor mechanism, which suffers from security risks when the attacker has obtained the password and smart card of the user. Furthermore, as the number of servers has increased remarkably to provide more services for the end user [25], a single-server architecture is unable to meet the needs of users. More registered servers will lead to more identities and passwords that the user must remember, which causes considerable database cost. Moreover, it is not secure for the users to use the same set of identities and passwords to register with different servers.

### 1.1. Main Contributions

To prevent an adversary from carrying out potential attacks, it is essential to design a robust authentication mechanism. In this paper, we propose a three-factor fast authentication scheme with time bound and user anonymity for multi-server e-health systems in 5G-based wireless sensor networks. Our scheme introduces three-factor authentication to address security issues of traditional authentications in e-health system. By means of the authentication protocol, the users must register with healthcare providers via a secure channel. After that, the users and the servers mutually authenticate and compute shared session keys via a public channel. Finally, the users can use these shared keys to get access to specific healthcare services. The contributions of our work can be summarized as follows.

- Three-factor authentication in the proposed protocol combines biometrics, password, and smart card for providing a high-security and privacy-preserving communication environment.
- Time-bound authentication helps in controlling user access, protecting sensitive information, and can be applied to many scenarios in healthcare such as access control to the users in WBANs, medical channel subscription, medical examination appointment, etc.
- Our work designs fast authentication to speed up the communication process.
- Our scheme is designed with multi-server architecture, which allows users to use a single password to obtain services from multiple servers. This advantage can simplify network workload and save a significant database cost.

### 1.2. Structure of the Paper

The rest of the paper is organized as follows. We present the literature review in Section 2. We briefly review Zhang et al.’s scheme [26] in Section 3. We describe system and security model in Section 4. We propose a three-factor authentication protocol with time bound and user anonymity for e-health systems in wireless body sensor networks in Section 5. Section 6 presents logical analysis of the proposed scheme using GNY logic. Section 7 presents verification proof of the proposed scheme using AVISPA tool. Section 8 presents semantic security analysis of our work. We present performance analysis of the proposed scheme in comparison with related works in Section 9. Section 10 presents implementation of the proposed scheme. Finally, some conclusions are given in Section 11.

## 2. Related Works

Today, the number of medical devices is increasing, making security problem in e-health cloud-based system more prominent. The associated security and privacy problems of the IoMT were presented in [27,28]. Besides, security and privacy issues in WSN for health and the environment have been addressed in serval reviews [29,30,31] and surveys [32,33,34]. Among the recently proposed cryptographic schemes for e-health systems, secure three-factor authentication mechanism [35,36,37] combining biometrics, password and smart card has recently attracted much attention.

Fan and Lin [38] proposed a three-factor authentication scheme based on biometrics. Their scheme can preserve the privacy of the biometric data of every user. Besides, Fan and Lin demonstrated the completeness of their proposed scheme with formal security analysis. Nevertheless, Fan and Lin’s scheme is susceptible to many well-known attacks, such as stolen-verifier attack, online password guessing attack, modification attack, impersonation attack, man-in-the-middle (MITM) attack, stolen smart card attack, desynchronization attack, and denial of service (DoS) attack. Moreover, Fan and Lin’s scheme cannot achieve user untraceability and requires a biometric data storage. Jiang et al. [39] proposed a robust privacy-preserving three-factor authentication protocol for e-health clouds. Remedying drawbacks in the predecessor scheme, Jiang et al. claimed that their proposed scheme can withstand various known attacks and provide more security features. However, we found that Jiang et al.’s scheme cannot resist replay attack, stolen smart card attack, desynchronization attack, and DoS attack. Recently, Zhang et al. [26] designed a dynamic authentication and three-factor key agreement with privacy protection for e-health. Although Zhang et al. stated that their scheme resists various well-known attacks, we found that Zhang et al.’s protocol is still vulnerable to DoS attack. Besides, Zhang et al.’s scheme suffers from storage burden of storing biometric data.

## 3. Review of Zhang et al.’s Scheme

Zhang et al. [26] designed a dynamic authentication and three-factor key agreement for the user and the server with privacy protection. Besides, the exact value of the biometric template remains unknown to the server. However, their scheme was found to have certain weaknesses. In this section, we present a brief review of Zhang et al.’s scheme and analyze its weaknesses.

### 3.1. Registration Phase

1. The user $U_{i}$ first enters his/her identity $ID_{i}$, password $PW_{i}$, and biometric template $B_{i}$, and then generates a random number string r. Next, the user $U_{i}$ computes $C_{1}=h\left(ID_{i}\left|\left|PW_{i}\right|\right|h_{Bio}\left(B_{i}\right)\right)$ and $C_{2}=B_{i}⊕r$. The user $U_{i}$ then transmits ($C_{1}$, $C_{2}$) as a registration request to the server via a secure channel.
2. After receiving ($C_{1}$, $C_{2}$), the server *S* uses private key k and $C_{2}$ to compute $M=h(h_{Bio}\;\left(C_{2}\right)||k)$. Then, the server S generates a random number string v, chooses $W_{0}=\mathrm{NULL}$, and calculates $W=h\left(h_{Bio}\;\left(C_{2}⊕v\right)\right)$, $X=h\left(ID_{SC}\left|\left|C_{1}\right|\right|M\right)⊕v$ and $Y=M⊕C_{1}$. The server S then stores {$C_{2}$, $W_{0}$, W} in database, and writes ($ID_{SC}$, h(.), $h_{Bio}$(.), X, Y) into smart card. After that, the server *S* sends the smart card to the user $U_{i}$ via a secure channel.
3. After receiving smart card from the server, the user $U_{i}$ computes $Z=r⊕h_{Bio}\left(B_{i}\right)$. Finally, the user $U_{i}$ stores Z in the smart card.

### 3.2. Login and Authentication Phase

1. The user $U_{i}$ uses $ID_{i}$, $PW_{i}$, $B_{i}$, and smart card to login to the server *S*, and then generates a random number string u. After that, the user $U_{i}$ calculates $C_{1}^{*}=h\left(ID_{i}\left|\left|PW_{i}\right|\right|h_{Bio}\left(B_{i}\right)\right)$, $M^{*}=Y⊕C_{1}^{*}$, $v^{*}=X⊕h\left(ID_{SC}\left|\left|C_{1}^{*}\right|\right|M^{*}\right)$, $r^{*}=Z⊕h_{Bio}\left(B_{i}\right)$, $C_{3}=h_{Bio}\left(B_{i}⊕r^{*}⊕v*\right)$, $C_{4}=B_{i}⊕r^{*}⊕h(M^{*}||u)$, and $C_{5}=u⊕h_{Bio}\left(B_{i}⊕r^{*}\right)$. Then, the user $U_{i}$ transmits ($C_{3}$, $C_{4}$, $C_{5}$) to the server S.
2. The server S computes $W^{*}=h\left(C3\right)$. After that, the server S searches $W^{*}$ in the dynamic verification table and obtains $C_{2}$. Otherwise, the medical server continues to search the column “dynamic string ($W^{0}$)” to see if a value is equal to $W^{*}$. If there is a match, the server S extracts the corresponding value $C_{2}$ and replaces W with the value of $W^{0}$. Otherwise, the medical server S rejects the login request. Next, the server S generates random number string β and calculates $M^{\prime }=h(h_{Bio}(C_{2}||k))$, $u^{*}=C_{5}⊕h_{Bio}\left(C_{2}\right)$, and $B_{i}⊕r^{*}=C_{4}⊕h(M^{\prime }||u^{*})$. Then, the server *S* checks if $B_{i}⊕r^{*}$ and $C_{2}$ are within a bearable threshold [40], then computes $C_{6}=\beta ⊕h\left(B_{i}⊕r^{*}\right)$ and $C_{7}=h\left(\left(B_{i}⊕r^{*}\right)\left|\left|u^{*}\right|\right|\beta \right)$. Next, the server S transmits ($C_{6}$, $C_{7}$) to the user $U_{i}$.
3. After receiving ($C_{6}$, $C_{7}$), the user $U_{i}$ computes $\beta_{*}=C_{6}⊕h\left(B_{i}⊕r^{*}\right)$. Next, the user $U_{i}$ checks if $C_{7}$ is equal to $h\left(\left(B_{i}⊕r^{*}\right)\left|\left|u\right|\right|\beta^{*}\right)$. If there is a match, the user $U_{i}$ compute $C_{8}=h\left(h_{Bio}\left(B_{i}⊕r^{*}⊕\beta^{*}\right)⊕\beta^{*}\right)$, $X_{new}=h\left(IDSC\left|\left|C_{1}^{*}\right|\right|M^{*}\right)⊕\beta^{*}$, and session key $SK=h\left(M^{*}\left|\left|u\right|\right|\beta^{*}\right)$. Thereafter, the user $U_{i}$ transmits $C_{8}$ to server S.
4. After receiving $C_{8}$, the server S compares $C_{8}$ with $h\left(h_{Bio}\left(B_{i}⊕r^{*}⊕\beta \right)⊕\beta \right)$. If there is a match, the server S accepts $SK=h\left(M^{\prime }\left|\left|u*\right|\right|\beta \right)$ as the session key. Next, the server *S* computes $W_{new}=h\left(h_{Bio}\left(C_{2}⊕\beta \right)\right)$. Then, the server S replaces ($W_{0}$, W) by (W, $W_{new}$) and calculates $C_{9}=h(SK||\beta )$. Then, the server S transmits $C_{9}$ to user $U_{i}$.
5. After receiving $C_{9}$, the user $U_{i}$ compares $C_{9}$ with $h(SK||\beta^{*})$. If there is a match, the user $U_{i}$ accepts SK as the session key. Finally, the user $U_{i}$ replaces X by $X_{new}$ in the smart card for the next login.

### 3.3. The Weaknesses

- Suffers from denial of service (DoS) attack: DoS attack is carried out by flooding the targeted host or network with traffic until the target cannot respond or simply crashes, preventing access for legitimate users [41]. In this case, timestamp solution is employed to verify the validity of the message. Without the timestamp included in login request message ($C_{3}$, $C_{4}$, $C_{5}$), Zhang et al.’s scheme is vulnerable to DOS attack.
- Suffers from a burden of biometric storage: The authentication based on biometric template requires a storage for storing biometric data. This additional storage does not make Zhang et al.’s scheme unsafe against insider attack since it does not consist of any information about passwords and the real biometric information in the database. However, it results in a significant cost that needs addressing.
- Lacks time-bound based access control: Time-based authentication is a good solution to prove an individual’s identity and authenticity on appearance simply by detecting its presence at a scheduled time of day. Lacking this feature in the work, Zhang et al.’s scheme is not well suited for e-health since time bound is useful in many cases, e.g., medical examination appointment.
- Lacks multi-server environment: Multi-server architecture allows user to obtain services from multiple servers using a single password, which greatly saves database cost. Without introducing multi-server architecture, communication in Zhang et al.’s scheme is not prominently efficient.

## 4. System and Security Model

### 4.1. System Model

**Figure 2:**
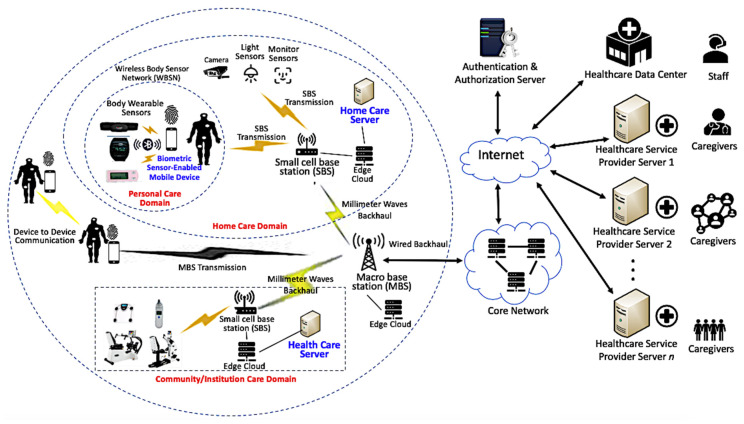
The proposed system model.

As shown in Figure 2, we propose a system model in which 5G-based smart healthcare network consists of various domains: community care domain, home care domain, and personal care domain. Sensors included in personal care domain are body wearable sensors and biometric sensor-enabled mobile device. They can provide a continuous health monitoring of a person without any constraint on his/her normal daily life activities [42]. Besides, home care domain includes some other sensors such as camera sensor, light sensor, etc. Community care domain includes temperature measuring sensor, sporting equipment, and other IoMT-enabled equipment.

Furthermore, within personal care domains, Wireless Body Sensor Network (WBSN) is a special case of the WBAN where all nodes in the network are sensors [43], which help in remotely collecting patient’s health record data (temperature, motion detection, sound, etc.) [31,44,45,46,47]. Besides, this patient can use mobile device to collect sensing data produced by his/her body wearable sensors. This monitoring system provides an interesting and widely accepted technology, obtaining special attention because of its friendly services in the smart world. In home care domains, the user may also use this mobile device to access other sensor-enabled devices through SBS transmission, thereby having comprehensive control of their home based on the authority of the home care server. Additionally, in 5G networks, user devices and MBSs can conduct direct transmission for healthcare services as long as they have spectrum opportunities. Furthermore, in community care environments, sensors and equipment are controlled by healthcare servers through SBSs. Thus, service providers can provide a continuity of care for the users.

In this system model, the user uses his/her mobile device and sensors to communicate with healthcare service provider and obtain specific services. Specifically, the user can login to home care server to query his/her own home care information. Besides, the user is able to upload his/her health data produced from wearable sensors to healthcare server. The user can also control light sensor, monitor sensor, and temperature measuring sensor from various healthcare domains. To accelerate the communication process, we design a fast authentication in the proposed scheme. The proposed scheme allows the communication between the user and the server to be carried out in a secure and privacy-preserved manner. Besides, Figure 2 also shows that our proposed multi-server environment allows the user to login to multiple healthcare service provider servers using a single password, thereby saving significant database cost and improving communication efficiency.

### 4.2. Security Model

Security risks in a public communication channel are common challenge for most of the wireless techniques. Data from the sensors and device in home domain are sensitive information and very likely to be compromised without a robust authentication mechanism. Besides, in home environment, data produced from these sensors are also very important and sensitive. For example, an adversary can impersonate the user to obtain the access to camera sensor, which strongly violates privacy of the user. In addition, in community care domain, sensor-enabled IoMT devices, for instance temperature measuring sensor, are likely vulnerable to security risks. The adversary may provide tampered information to the server after compromising these sensors.

Specifically, various attacks threatening the network access legitimacy are described as follows. *MITM attacks* is when the attacker compromises the transmitted message while the sender and the receiver believe that they are directly communicating with each other. *Impersonation attacks* happen when the attacker has obtained the identity of a user, and then attempts to impersonate him/her. *Replay attacks* let a malicious attacker intercept messages from the last communication session to derive the session key. In addition, the importance of user privacy protection in online communication is prominent [48,49,50]. Solving the contradiction between user anonymity and authentication is still a big challenge in this research area.

For the security of the proposed scheme, the following essential requirements should be met to ensure a secure and privacy-preserved communication between the user and the server.

- Mutual authentication: Only the user with valid registered information can be successfully authenticated and is able to compute an exact session key to obtain service provided by the server. On the other hand, the server must be also authenticated as a legitimate party to provide true information for the user.
- Session key establishment: The purpose of this work is to allow the user and the server to securely negotiate a session key for the communication between them.
- User anonymity: We expect privacy of the user can be preserved during communication process.
- Biometric template anonymity: Three-factor mechanism includes biometric template in registration and authentication process. Our purpose is to not allow user’s biometric template to be revealed to the public.
- Forward secrecy: Our work aims to prevent the attacker from using information from the past communication session to derive the key.

## 5. The Proposed Scheme

Our proposed scheme includes two roles: user $U_{i}$ and server $S_{j}$. The purpose of the proposed protocol is to allow the user $U_{i}$ and the server $S_{j}$ to compute a shared session key in a secure and privacy-preserved manner. The user $U_{i}$ first must register with the server $S_{j}$ as a legitimate party. Next, the user $U_{i}$ and the user $S_{j}$ mutually authenticate based on their information, and then compute a session key via a public channel. The authentication process consists of four phases: initialization phase, registration phase, login and initial authentication phase, and fast authentication phase. Table 1 describes notations and cryptographic functions used in this paper.

### 5.1. Initialization Phase

Our work employs Rabin cryptosystem [51], encryption process of which is extremely fast and easy (as long as encryption does not require computing a Jacobi symbol), while decryption of which (using the Chinese remainder theorem) is roughly of the same speed as RSA decryption. In this phase, based on Rabin cryptosystem, initial parameters are generated to carry out whole authentication process.

1. Server: The server $S_{j}$ chooses two arbitrary big numbers ($p_{j}$, $q_{j}$), then compute $n_{j}=p_{j}$ · $q_{j}$, which satisfies $p_{j}\equiv q_{j}\equiv 3\;\left(mod\;4\right)$, where $p_{j}$ and $q_{j}$ are private keys, and $n_{j}$ is public key of the server $S_{j}$. The server $S_{j}$ then randomly selects a string $x_{j}$ as the symmetric encryption key of the server $S_{j}$. The server $S_{j}$ then secretly stores ($p_{j}$, $q_{j}$, $x_{j}$).
2. Smart card: The user has the smart card choose and store a random string σ.

### 5.2. Registration Phase

**Figure 3:**
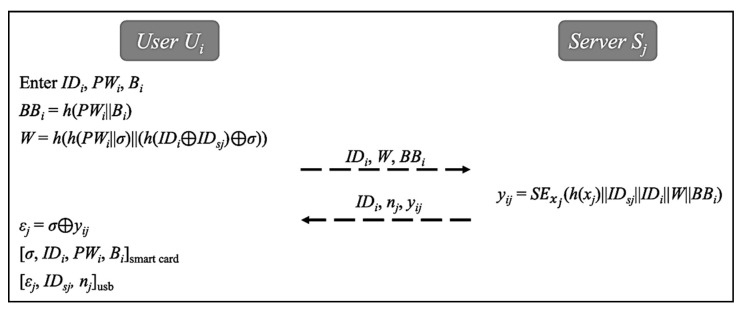
Registration phase.

Before using the service, the user $U_{i}$ must register with the server $S_{j}$ via a secure channel. In this phase, the information of the user and the server are secretly stored. For that purpose, both sides perform the following steps to complete the registration phase. The procedure is shown in Figure 3.

1. The user $U_{i}$ first enters identity $ID_{i}$, password $PW_{i}$ and biometric template $B_{i}$, then computes $BB_{i}=h(PW_{i}||B_{i})$ and $W=h(h(PW_{i}||\sigma )||\left(h\left(ID_{i}⊕ID_{S_{j}}\right)⊕\sigma \right))$. Next, the user $U_{i}$ transmits $ID_{i}$, W and $BB_{i}$ to the sever $S_{j}$.
2. After receiving message ($ID_{i}$, W, $BB_{i}$), the server $S_{j}$ uses symmetric encryption key $x_{j}$ to compute $y_{ij}=SE_{x_{j}}\left(h\left(x_{j}\right)\left|\left|\;ID_{S_{j}}\right|\right|ID_{i}\left|\left|W\right|\right|BB_{i}\right)$. Thereafter, the server $S_{j}$ transmits ($ID_{i}$, $n_{j}$, $y_{ij}$) to the user $U_{j}$.

After receiving the message, the user $U_{i}$ computes $\varepsilon_{j}=\sigma ⊕y_{ij}$. The user $U_{i}$ then stores (σ, $ID_{i}$, $PW_{i}$, $B_{i}$) and ($\varepsilon_{j}$, $ID_{S_{j}}$, $n_{j}$) into smart card and flash drive, respectively.

### 5.3. Login and Initial Authentication Phase

**Figure 4:**
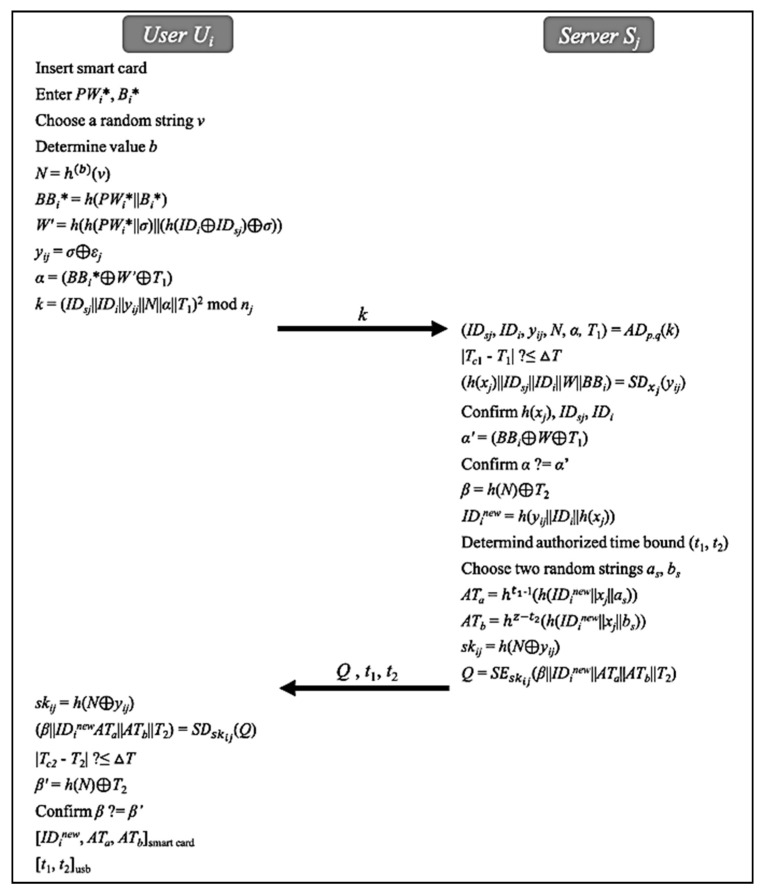
Login and initial authentication phase.

If the user $U_{i}$ wants to use service from healthcare provider, he/she has to communicate with the sever $S_{j}$ to calculate a session key. Since this communication is carried out via a public channel, an authentication procedure is required to ensure they are legitimate parties. As shown in Figure 4, the user $U_{i}$ and the server $S_{j}$ perform the following steps to complete login and initial authentication phase.

1. The user $U_{i}$ first inserts the smart card and enter $PW_{i}^{*}$ and $B_{i}^{*}$. Next, the user $U_{i}$ chooses a random string v, determines the number of authentications b, and computes $N=h^{\left(b\right)}\left(v\right)$, $BB_{i}^{*}=h(PW_{i}^{*}||B_{i}^{*})$, $W^{\prime }=h(h(PW_{i}^{*}||\sigma )||\left(h\left(ID_{i}⊕ID_{S_{j}}\right)⊕\sigma )\right)$, $y_{ij}=\sigma ⊕\varepsilon_{j}$, $\alpha =\left(BB_{i}^{*}⊕W^{\prime }⊕T_{1}\right)$, and $k={\left(ID_{S_{j}}\left|\left|ID_{i}\right|\right|y_{ij}\left|\left|N\right|\right|\alpha ||T_{1}\right)}^{2}\;mod\;n_{j}$. Then, the user $U_{i}$ transmits k to the server $S_{j}$.
2. After receiving k, the server $S_{j}$ uses private keys $p_{j}$, $q_{j}$ to decrypt *k* then confirms the validity of the timestamp $T_{1}$. Next, it uses symmetric key $x_{j}$ to decrypt $y_{ij}$ obtained from k. The server $S_{j}$ then verifies $h\left(x_{j}\right)$, $ID_{i}$ and $ID_{S_{j}}$. Thereafter, the server $S_{j}$ computes $\alpha^{\prime }=\left(BB_{i}⊕W⊕T_{1}\right)$. The server $S_{j}$ then compares α with $\alpha^{\prime }$. If there is a match, the server $S_{j}$ calculates $\beta =h\left(N\right)⊕T_{2}$ and new identity $ID_{i}^{new}=h\left(y_{ij}\left|\left|ID_{i}\right|\right|\;h\left(x_{j}\right)\right)$. The server $S_{j}$ then determines the time bound (*t*_1_, *t*_2_), and choose two random strings $a_{s}$ and $b_{s}$. Next, the server $S_{j}$ computes $AT_{a}=h^{t_{1}-1}\left(h\left(ID_{i}^{new}\left|\left|x_{j}\right|\right|a_{s}\right)\right)$, $AT_{b}=h^{z-t_{2}}\left(h\left(ID_{i}^{new}\left|\left|x_{j}\right|\right|b_{s}\right)\right)$, session key $sk_{ij}=h\left(N⊕y_{ij}\right)$ and $Q=SE_{sk_{ij}}\left(\beta \left|\left|ID_{i}^{new}\left|\left|AT_{a}\right|\right|AT_{b}\right|\right|T_{2}\right)$. Then, the server $S_{j}$ transmits (Q, *t*_1_, *t*_2_) to the user $U_{i}$.
3. After receiving (Q, *t*_1_, *t*_2_), the user $U_{i}$ computes $sk_{ij}=h\left(N⊕y_{ij}\right)$. Next, the user $U_{i}$ uses session key $sk_{ij}$ to decrypt Q and confirms the validity of the timestamp $T_{2}$. Thereafter, the user $U_{i}$ computes $\beta^{\prime }=h\left(N\right)⊕T_{2}$ and confirms β. If there is a match the user $U_{i}$ accepts session key $sk_{ij}$. Finally, the user $U_{i}$ stores ($ID_{i}^{new}$, $AT_{a}$, $AT_{b}$) and (*t*_1_, *t*_2_) in the smart card and flash drive, respectively.

### 5.4. Fast Authentication Phase

**Figure 5:**
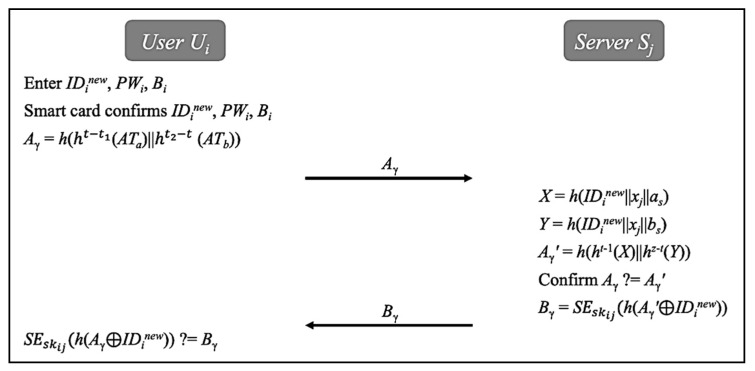
Fast authentication phase.

As stated above, we design the fast authentication in our work to accelerate communication process. After the initial authentication, the user $U_{i}$ and the server $S_{j}$ are allowed to quickly authenticate each other based on an authorized time bound without computing a new session key. As shown in Figure 5, both sides perform the following steps to complete the fast authentication.

1. The user $U_{i}$ enters $ID_{i}^{new}$, $PW_{i}$ and $B_{i}$. The smart card confirms $ID_{i}^{new}$, $PW_{i}$, and $B_{i}$. Next, the user $U_{i}$ computes $A_{\gamma }=h(h^{t-t_{1}}\left(AT_{a}\right)||h^{t_{2}-t}\left(AT_{b}\right))$. Then, the user $U_{i}$ transmits $A_{\gamma }$ to the server $S_{j}$.
2. After receiving $A_{\gamma }$, the server $S_{j}$ calculates $X=h\left(ID_{i}^{new}\left|\left|x_{j}\right|\right|a_{s}\right)$, $Y=h\left(ID_{i}^{new}\left|\left|x_{j}\right|\right|b_{s}\right)$ and $A_{\gamma }^{\prime }=h(h^{t-1}\left(X\right)||h^{z-t}\left(Y\right))$. Next, the server $S_{j}$ compares $A_{\gamma }$ with $A_{\gamma }^{\prime }$. If there is no match, the server $S_{j}$ will revoke the session key $sk_{ij}$; otherwise, it computes $B_{\gamma }=SE_{sk_{ij}}\left(h\left(A_{\gamma }^{\prime }⊕ID_{i}^{new}\right)\right)$. The server $S_{j}$ then transmits $B_{\gamma }$ to the user $U_{i}$.
3. After receiving $B_{\gamma }$, the user $U_{i}$ computes $SE_{sk_{ij}}\left(h\left(A_{\gamma }⊕ID_{i}^{new}\right)\right)$, and then compares it with $B_{\gamma }$. If there is a match, the user $U_{i}$ accepts $sk_{ij}$. Following this, the user $U_{i}$ can still use the session key $sk_{ij}$ to obtain the healthcare service in this communication session.

## 6. Logical Analysis Using GNY Logic

In this section, we prove security completeness and correctness of our proposed protocol through logical roles of GNY (Gong–Needham–Yahalom) logic [52]. GNY logic has been widely used to formally analyze the completeness of a cryptographic protocol. The proposed scheme is presented in logic as follows.

Message *k*

$U_{i}$ → $S_{j}$: ({$ID_{S_{j}}$, $ID_{i}$, {*H*($x_{j}$), $ID_{S_{j}}$, $ID_{i}$, *H*(*H*($PW_{i}$, σ), *F*(*H*(*F*($ID_{i}$, $ID_{S_{j}}$)), σ)), *H*($PW_{i}$, $B_{i}$)${\}}_{x_{j}}$, $H^{\left(b\right)}$(*v*), *F*(*H*($PW_{i}^{*}$, $B_{i}^{*}$), *H*(*H*($PW_{i}^{*}$, σ), *F*(*H*(*F*($ID_{i}$, $ID_{S_{j}}$)), σ)), $T_{1}$), $T_{1}{\}}_{\mathrm{mod}\;n_{j}}$)

Message (Q, *t*_1_, *t*_2_)

$S_{j}$ → $U_{i}$: ({*F*(*H*(*N*), $T_{2}$), *H*({*H*($x_{j}$), $ID_{S_{j}}$, $ID_{i}$, *H*(*H*($PW_{i}$, σ), *F*(*H*(*F*($ID_{i}$, $ID_{S_{j}}$)), σ)), *H*($PW_{i}$, $B_{i}$)${\}}_{x_{j}}$, $ID_{i}$, *H*($x_{j}$)), $H^{t_{1}-1}$(*H*(*H*({*H*($x_{j}$), $ID_{S_{j}}$, $ID_{i}$, *H*(*H*($PW_{i}$, σ), *F*(*H*(*F*($ID_{i}$, $ID_{S_{j}}$)), σ)), *H*($PW_{i}$, $B_{i}$)${\}}_{x_{j}}$, $ID_{i}$, *H*($x_{j}$)), $x_{j}$, $a_{s}$)), $H^{z-t_{2}}$(*H*(*H*({*H*($x_{j}$), $ID_{S_{j}}$, $ID_{i}$, *H*(*H*($PW_{i}$, σ), *F*(*H*(*F*($ID_{i}$, $ID_{S_{j}}$)), σ)), *H*($PW_{i}$, $B_{i}$)${\}}_{x_{j}}$, $ID_{i}$, *H*($x_{j}$)), $x_{j}$, $b_{s}$)), $T_{2}{\}}_{sk_{ij}}$, *t*_1_, *t*_2_)

### 6.1. Logical Rules Used in Our Proof

- (I_1_) $\frac{P⊲*{\left\{X\right\}}_{K},\;\;\;P∋K,\;\;\;P|\equiv P\overset{K}{↔}Q,\;\;\;P|\equiv \emptyset (X),\;\;\;P|\equiv \#(X,\;\;\;K)}{P|\equiv Q|∼X,\;\;\;P|\equiv Q|∼{\left\{X\right\}}_{K},\;\;\;P|\equiv Q∋K}$: Suppose that for princial *P* all of the following conditions hold: (1) *P* receives a formula consisting of a *X* encrypted with key *K* and marked with a not-originated-here mark; (2) *P* possesses *K*; (3) P believes *K* is a suitable secret for himself and *Q*; (4) *P* believes formula *X* is recognizable; and (5) *P* believes that *K* is fresh or that *X* is fresh. Then, *P* is entitled to believe that: (1) *Q* once conveyed *X*; (2) *Q* once conveyed the formula *X* encrypted with *K*; and (3) Q possesses *K*.
- (I_2_) $\frac{P⊲*{\left\{X,\;\;\;\langle S\rangle \right\}}_{+K},\;\;\;P∋\left(-K,\;\;\;S\right),\;\;\;P|\equiv \overset{+K}{\to }P,\;\;\;P|\equiv P\overset{S}{↔}Q,\;\;\;P|\equiv \emptyset \left(X,S\right),\;\;\;P|\equiv \#\left(X,\;\;\;S,\;+K\right)}{P|\equiv Q|∼(X,\;\;\langle S\rangle ),\;\;\;P|\equiv Q|∼{\left\{X,\;\;\langle S\rangle \right\}}_{+K},\;\;\;P|\equiv Q∋+K}$: Suppose that for principal *P* all of the following conditions hold: (1) *P* receives a formula consisting of *X* concatenated with *S*, encrypted with a public key, and marked with a not-originated-here mark; (2) *P* possesses *S* and the corresponding private key; (3) *P* believes the public key is his own; (4) *P* believes S is a suitable secret for himself and *Q*; (5) *P* believes that *X* concatenated with S is recognizable; and (6) *P* believes that at least one of *S*, *X*, or +*K* is fresh. Then, *P* is entitled to believes that: (1) *Q* once conveyed the formula *X* concatenated with S; (2) *Q* once conveyed the formula *X* concatenated with *S* and encrypted with the public key; and (3) *Q* possesses the public key.
- (I_7_) $\frac{P\left|\equiv Q\right|∼\left(X,\;\;\;Y\right)}{P\left|\equiv Q\right|∼X}$: *P* believes *Q* once conveyed a formula consisting of *X*, and then *P* is entitled to believe *Q* once conveyed *X*.
- (J_1_) $\frac{P\left|\equiv Q\right|\Rightarrow C,\;\;\;P\left|\equiv Q\right|\equiv C}{P|\equiv C}$: *P* believes that *Q* is an authority on some statement *C* and that *Q* believes in *C*, and then *P* should believe in *C* as well.
- (F_1_) $\frac{P|\equiv \#\left(X\right)}{P|\equiv \#(X,\;\;\;Y),\;\;\;P|\equiv \#\left(F\left(X\right)\right)}$: *P* believes message *X* is fresh, which means *P* can believe that any (*X*, *Y*) including message *X* is fresh, and then *P* believes *F*(*X*), which is computed from message *X*, is also fresh.
- (T_1_) $\frac{P⊲*X}{P⊲X}$: When *P* obtains a non-original value *X, it means *P* may obtain the original *X*.
- (T_3_) $\frac{P⊲{\left\{X\right\}}_{K},\;\;\;P∋K}{P⊲X}$: *P* uses secret key *K* to encrypt, decrypt to obtain message *X*.
- (T_4_) $\frac{P⊲{\left\{X\right\}}_{+K},\;\;\;P∋-K}{P⊲X}$: *P* uses private key −*K* to decrypt, uses public key +K to encrypt, and obtains the message *X*.
- (P_1_) $\frac{P⊲X}{P∋X}$: *P* can see the message *X*, indicating that *P* really possesses the message *X*.
- (P_4_) $\frac{P∋X}{P∋H\left(X\right)}$: If *P* possesses *X*, then it possesses *H*(*X*).
- (R_1_) $\frac{P|\equiv \emptyset \left(X\right)}{P|\equiv \emptyset (X,\;\;\;Y),\;\;\;P|\equiv \emptyset \left(F\left(X\right)\right)}$: *P* believes message *X* is recognizable, indicating that *P* can believe that any (*X*, *Y*) including message *X* is recognizable, and *P* believes that any *F*(*X*) computed from message *X* is also recognizable).
- (R_2_) $\frac{P|\equiv \emptyset \left(X\right),\;\;\;P∋K}{P|\equiv \emptyset ({\left\{X\right\}}_{K}),\;\;\;P|\equiv \emptyset \left({\left\{X\right\}}_{K}^{-1}\right)}$: *P* believes message *X* is recognizable and *P* possesses the shared secret key *K*, and then *P* believes anything computed using the shared secret key is recognizable.
- (R_4_) $\frac{P|\equiv \emptyset \left(X\right),\;\;\;P∋-K}{P|\equiv \emptyset \left({\left\{X\right\}}_{-K}\right)}$: *P* believes the message *X* is recognizable and *P* possesses private key −*K*, then *P* believes any message computed using private key is recognizable.

### 6.2. Assumptions of the Proposed Protocol

- (A_1_) $S_{j}$ ϶ $p_{j}$, $q_{j}$: The server $S_{j}$ possesses private keys $p_{j}$ and $q_{j}$.
- (A_2_) $S_{j}$ ϶ $x_{j}$: The server $S_{j}$ possesses secret key $x_{j}$.
- (A_3_) $S_{j}$ ϶ *N*: The server $S_{j}$ possesses message *N*.
- (A_4_) $S_{j}$ |≡ ∅(*H*($x_{j}$)): The server $S_{j}$ believes that *H*($x_{j}$) is recognizable.
- (A_5_) $S_{j}$ |≡ ∅(*α*): The server $S_{j}$ believes that *α* is recognizable.
- (A_6_) $U_{i}$ |≡ #(*T*): The user *U_i_* believes that timestamp *T* is fresh.
- (A_7_) $S_{j}$ |≡ ($U_{i}$ $\overset{N}{↔}$ $S_{j}$): The server $S_{j}$ believes that *N* is a suitable secret for the user $U_{i}$ and the server $S_{j}$.
- (A_8_) $U_{i}$ ϶ *N*: The user $U_{i}$ possesses *N*.
- (A_9_) $U_{i}$ ϶ $y_{ij}$: The user $U_{i}$ possesses the key $y_{ij}$.
- (A_10_) $U_{i}$ |≡ ∅(*v*): The user $U_{i}$ believes that *v* is recognizable.
- (A_11_) $S_{j}$ |≡ $U_{i}$ |⇒ ($U_{i}$ $\overset{N}{↔}$ $S_{j}$): The server $S_{j}$ believes that the user *U_i_* has jurisdiction over *N*, which is a suitable secret for the user $U_{i}$ and the server $S_{j}$.
- (A_12_) $S_{j}$ |≡ #(*T*): The server $S_{j}$ believes that timestamp *T* is fresh.

### 6.3. Goals

- Message content authentication: It proves the authenticity of transmitted message.

Goal 1: Prove the authenticity of message *k*:

(G1) $$S_{j}\;|\equiv \;\emptyset \;(\left\{ID_{S_{j}},\;ID_{i},\;{\left\{H(x_{j}),\;ID_{S_{j}},\;ID_{i},\;H(H(PW_{i},\;\sigma ),\;F(H(F(ID_{i},\;ID_{S_{j}})),\;\sigma )),\;H(PW_{i},\;B_{i})\right\}}_{x_{j}},\;\;H^{\left(b\right)}(v),\;F(H(PW_{i}^{*},\;B_{i}^{*}),\;H(H(PW_{i}^{*},\;\sigma ),\;F(H(F(ID_{i},\;ID_{S_{j}})),\;\sigma )),\;T_{1}),\;T_{1}\right\}{}_{\mathrm{mod}\;n_{j}})$$

Only the server $S_{j}$ can read message *k* transmitted by the user $U_{i}$.

Goal 2: Prove the authenticity of the message (Q, *t*_1_, *t*_2_):

(G2) $$U_{i}\;|\equiv \;\emptyset \;(\{F(H(N),\;T_{2}),\;H({\left\{H(x_{j}),\;ID_{S_{j}},\;ID_{i},\;H(H(PW_{i},\;\sigma ),\;F(H(F(ID_{i},\;ID_{S_{j}})),\;\sigma )),\;H(PW_{i},\;B_{i})\right\}}_{x_{j}},\;\;ID_{i},\;H(x_{j})),\;H^{t_{1}-1}(H(H({\left\{H(x_{j}),\;ID_{S_{j}},\;ID_{i},\;H(H(PW_{i},\;\sigma ),\;F(H(F(ID_{i},\;ID_{S_{j}})),\;\sigma )),\;H(PW_{i},\;B_{i})\right\}}_{x_{j}},\;\;ID_{i},\;H(x_{j})),\;x_{j},\;a_{s})),\;H^{z-t_{2}}(H(H({\left\{H(x_{j}),\;ID_{S_{j}},\;ID_{i},\;H(H(PW_{i},\;\sigma ),\;F(H(F(ID_{i},\;ID_{S_{j}})),\;\sigma )),\;H(PW_{i},\;B_{i})\right\}}_{x_{j}},\;\;ID_{i},\;H(x_{j})),\;x_{j},\;b_{s})),\;T_{2}{\}}_{sk_{ij}},\;t_{1},\;t_{2})$$

Only the user $U_{i}$ can read message (Q, *t*_1_, *t*_2_) transmitted by the server $S_{j}$.

- Message origin authentication: It proves that the received message is transmitted by the legitimate parties.

Goal 3: Prove the origin of message k:

(G3) $$S_{j}\;|\equiv \;U_{i}\;|\sim \;(\{ID_{S_{j}},\;ID_{i},\;{\left\{H(x_{j}),\;ID_{S_{j}},\;ID_{i},\;H(H(PW_{i},\;\sigma ),\;F(H(F(ID_{i},\;ID_{S_{j}})),\;\sigma )),\;H(PW_{i},\;B_{i})\right\}}_{x_{j}},\;\;H^{\left(b\right)}(v),\;F(H(PW_{i}^{*},\;B_{i}^{*}),\;H(H(PW_{i}^{*},\;\sigma ),\;F(H(F(ID_{i},\;ID_{S_{j}})),\;\sigma )),\;T_{1}),\;T_{1}{\}}_{\mathrm{mod}\;n_{j}})$$

The server $S_{j}$ can verify that only the user $U_{i}$ can generate message k received by the server $S_{j}$.

Goal 4: Prove the origin of message (Q, *t*_1_, *t*_2_):

(G4) $$U_{i}\;|\equiv \;S_{j}\;|\sim \;(\{F(H(N),\;T_{2}),\;H({\left\{H(x_{j}),\;ID_{S_{j}},\;ID_{i},\;H(H(PW_{i},\;\sigma ),\;F(H(F(ID_{i},\;ID_{S_{j}})),\;\sigma )),\;H(PW_{i},\;B_{i})\right\}}_{x_{j}},\;\;ID_{i},\;H(x_{j})),\;H^{t_{1}-1}(H(H({\left\{H(x_{j}),\;ID_{S_{j}},\;ID_{i},\;H(H(PW_{i},\;\sigma ),\;F(H(F(ID_{i},\;ID_{S_{j}})),\;\sigma )),\;H(PW_{i},\;B_{i})\right\}}_{x_{j}},\;\;ID_{i},\;H(x_{j})),\;x_{j},\;a_{s})),\;H^{z-t_{2}}(H(H({\left\{H(x_{j}),\;ID_{S_{j}},\;ID_{i},\;H(H(PW_{i},\;\sigma ),\;F(H(F(ID_{i},\;ID_{S_{j}})),\;\sigma )),\;H(PW_{i},\;B_{i})\right\}}_{x_{j}},\;\;ID_{i},\;H(x_{j})),\;x_{j},\;b_{s})),\;T_{2}{\}}_{sk_{ij}},\;t_{1},\;t_{2})$$

The user $U_{i}$ can verify that only the server $S_{j}$ can generate message (Q, *t*_1_, *t*_2_) received by the user $U_{i}$.

- Key agreement and confirmation: They prove that the session key is secret and shared only by the legitimate parties.

Goal 5: Key Agreement of $U_{i}$ → $S_{j}$: 

(G5) $$U_{i}\;|\equiv \;S_{j}\;϶\;sk_{ij}$$

The user $U_{i}$ believes that only the server $S_{j}$ can obtain the shared session key $sk_{ij}$.

Goal 6: Key Confirmation of $U_{i}$ → $S_{j}$: 

(G6) $$U_{i}\;|\equiv \;S_{j}\;|\equiv \;(U_{i}\;\overset{sk_{ij}}{↔}\;S_{j})$$

The user $U_{i}$ believes that the user server $S_{j}$ is convinced of the shared session key $sk_{ij}$ established between them.

Goal 7: Key Agreement of $S_{j}$ → $U_{i}$: 

(G7) $$S_{j}\;|\equiv \;(U_{i}\overset{sk_{ij}}{↔}\;S_{j})$$

The server $S_{j}$ believes that a shared session key $sk_{ij}$ between it and the user $U_{i}$ has been established.

Goal 8: Key Confirmation of $S_{j}$ → $U_{i}$: 

(G8) $$S_{j}\;|\equiv \;U_{i}\;|\equiv \;(U_{i}\overset{sk_{ij}}{↔}\;S_{j})$$

The server $S_{j}$ believes that the user $U_{i}$ has already obtained the shared session key $sk_{ij}$.

Since $S_{j}$ knows of message *k*, we have that:

(1) $$S_{j}\;⊲\;*\left(*{\left\{ID_{S_{j}},\;ID_{i},\;y_{ij},\;N,\;\alpha ,\;T_{1}\right\}}_{\mathrm{mod}\;n_{j}}\right)$$

According to T_1_, we have that:

(2) $$S_{j}\;⊲\;\left({\left\{ID_{S_{j}},\;ID_{i},\;y_{ij},\;N,\;\alpha ,\;T_{1}\right\}}_{\mathrm{mod}\;n_{j}}\right)$$

According to Equation (2), A_1_, and T_4_, the server $S_{j}$ can use private keys $p_{j}$ and $q_{j}$ to decrypt *k*; we have that:

(3) $$S_{j}\;⊲\;(ID_{S_{j}},\;ID_{i},\;y_{ij},\;N,\;\alpha ,\;T_{1})$$

According to Equation (3), A_2_, and T_3_, the server $S_{j}$ can use secret key $x_{j}$ to decrypt $y_{ij}$ = {*H*($x_{j}$), $ID_{S_{j}}$, $ID_{i}$, *H*(*H*($PW_{i}$, *σ*), *F*(*H*(*F*($ID_{i}$, *I*$ID_{S_{j}}$)), *σ*)), *H*($PW_{i}$, $B_{i}$)${\}}_{x_{j}}$; we have that:

(4) $$S_{j}\;⊲\;(ID_{S_{j}},\;ID_{i},\;H(x_{j}),\;ID_{S_{j}},\;ID_{i},\;H(H(PW_{i},\;\sigma ),\;F(H(F(ID_{i},\;ID_{S_{j}})),\;\sigma )),\;H(PW_{i},\;B_{i}),\;H^{\left(b\right)}(v),\;F(H(PW_{i}^{*},\;B_{i}^{*}),\;H(H(PW_{i}^{*},\;\sigma ),\;F(H(F(ID_{i},\;ID_{S_{j}})),\;\sigma )),\;T_{1}),\;T_{1}))$$

According to (4) and P_1_, we have that:

(5) $$S_{j}\;϶\;ID_{i},\;ID_{S_{j}},\;H(x_{j}),\;F(H(PW_{i}^{*},\;B_{i}^{*}),\;H(H(PW_{i}^{*},\;\sigma ),\;F(H(F(ID_{i},\;ID_{S_{j}})),\;\sigma )),\;T_{1}))$$

According to (5), A_4_, A_5_, and R_1_, we have that:

(6) $$S_{j}\;|\equiv \;\emptyset \;(ID_{i},\;ID_{S_{j}},\;H(x_{j}),\;F(H(PW_{i}^{*},\;B_{i}^{*}),\;H(H(PW_{i}^{*},\;\sigma ),\;F(H(F(ID_{i},\;ID_{S_{j}})),\;\sigma )),\;T_{1})))$$

According to Equation (6), $S_{j}$ can believe *H*($x_{j}$) is truly recognizable. According to A_2_ and R_4_, the server $S_{j}$ possesses $x_{j}$ and can identify $H\left(x_{j}\right)$. Therefore, the server $S_{j}$ believes $y_{ij}$ (encrypted using $x_{j}$) is recognizable. We have that:

(7) $$S_{j}\;|\equiv \;\emptyset \;(y_{ij})\;⟺\;S_{j}\;|\equiv \;\emptyset ({\left\{H(x_{j}),\;ID_{S_{j}},\;ID_{i},\;H(H(PW_{i},\;\sigma ),\;F(H(F(ID_{i},\;ID_{S_{j}})),\;\sigma )),\;H(PW_{i},\;B_{i})\right\}}_{x_{j}})$$

According to Equations (6) and (7), A_5_, and R_1_, (G1) is realized by our protocol.

Since the user $U_{i}$ knows of message (Q, *t*_1_, *t*_2_), we have that:

(8) $$U_{i}\;⊲\;*(*\{\beta ,\;ID_{i}^{new},\;AT_{a},\;AT_{b},\;T_{2}{\}}_{sk_{ij}},\;t_{1},\;t_{2})$$

Based on rule T_1_, we have that:

(9) $$U_{i}\;⊲\;({\left\{\beta ,\;ID_{i}^{new},\;AT_{a},\;AT_{b},\;T_{2}\right\}}_{sk_{ij}},\;t_{1},\;t_{2})$$

Based on A_8_ and A_9_, we have that:

(10) $$U_{i}\;϶\;F(N,\;y_{ij})$$

Based on (10) and rule P_4_, the user $U_{i}$ can possess the shared secret key $sk_{ij}$; we have that:

(11) $$U_{i}\;϶\;H(F(N,\;y_{ij}))\;⟺\;U_{i}\;϶\;sk_{ij}$$

Based on Equations (9) and (11), and rule T_3_, *U_i_* can use the shared key $sk_{ij}$ to decrypt *Q* = {β, $ID_{i}^{new}$, $AT_{a}$, $AT_{b}$, $T_{2}{\}}_{sk_{ij}}$; we have that:

(12) $$U_{i}\;⊲\;(\beta )\;⟺\;U_{i}\;⊲\;F(H(N),\;T_{2})\;⟺\;U_{i}\;⊲\;F(H(H^{\left(b\right)}(v)),\;T_{2})$$

Based on Equation (12), A_10_, and rule R_1_, we have that:

(13) $$U_{i}\;|\equiv \;\emptyset (H^{\left(b\right)}(v))\;⟺\;U_{i}\;|\equiv \;\emptyset (N)$$

Based on Equation (13) and R_1_, we have that:

(14) $$U_{i}\;|\equiv \;\emptyset (F(H(N),\;T_{2}))\;⟺\;U_{i}\;|\equiv \;\emptyset (\beta )$$

Based on Equations (11) and (14), and rule R_2_, *U_i_* can possess $sk_{ij}$ and identify β. Since the user $U_{i}$ believes message *Q* encrypted using $sk_{ij}$ is recognizable, (Q, *t*_1_, *t*_2_) is truly the message which is encrypted using $sk_{ij}$ possessed by the user $U_{i}$. Hence, the proposed scheme realizes (G2).

According to (1), (3), A_4_, A_5_, A_12_, F_1_, and I_2_, (G3) is achieved.

Based on Equations (8), (10) and (11), A_3_, A_6_, F_1_, and I_1_, (G4) is achieved.

Based on (G4) and rule I_7_, our scheme realizes (G5).

Since the users $U_{i}$ believes the server $S_{j}$ is legitimate and has jurisdiction, we have $U_{i}$ |≡ $S_{j}$ |⇒ $S_{j}$ |≡ *. Based on (G4), (G6) is realized.

Based on (G3), A_11_, and rule J_1_, (G7) is realized.

Since the server $S_{j}$ believes the user $U_{i}$ is legitimate and has jurisdiction, we have $S_{j}$ |≡ $U_{i}$ |⇒ $U_{i}$ |≡ *. Based on (G3), our proposed scheme realizes (G8).

## 7. Security Analysis Using AVISPA Tool

### 7.1. Overview of AVISPA

**Figure 6:**
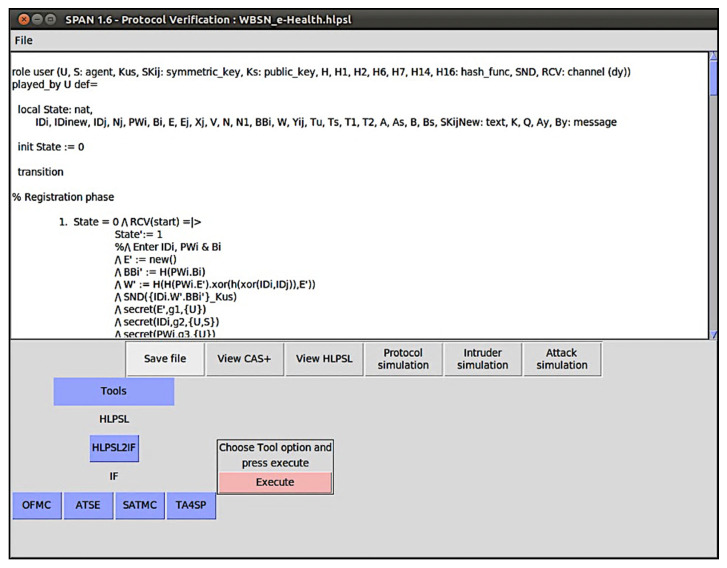
Security Protocol Animator for AVISPA.

Automated Validation of Internet Security Protocols and Applications (AVISPA) [53] is a widely accepted tool used for the analysis of large-scale Internet security-sensitive protocols and applications. AVISPA tool executes the simulated protocol specified by HLPSL language [54]. For verifying cryptographic protocol, AVISPA tool includes four backends: On-the-fly Model-Checker (OFMC), Constraint Logic based Attack Searcher (CL-AtSe), SAT-based ModelChecker (SATMC), and Tree Automata based on automatic approximations for the analysis of security protocols (TA4SP). In this paper, using AVISPA tool and Security Protocol Animator (SPAN), we provide a security proof for the proposed scheme. Figure 6 shows the interface of the SPAN with AVISPA tool.

### 7.2. The Verification

The proposed protocol is verified using the OFMC and CL-AtSe backends. In AVISPA, our scheme incudes two roles: user U and server S. The HLPSL specifications of the user U and the sever S are shown in Box 1 and Box 2, respectively. Besides, session role, environment role and goals are also specified in HLPSL, as shown in Box 3. For verification of the proposed scheme, we consider seven secrecy goals and three authentication goals as follows.

- secrecy_of g1: E’ is kept secret to the user U.
- secrecy_of g2: IDi is kept secret to the user U and the server S.
- secrecy_of g3: PWi is kept secret to the user U.
- secrecy_of g4: Bi is kept secret to the user U.
- secrecy_of g5: Xj is kept secret to the server S.
- secrecy_of g6: As’ is kept secret to the server S.
- secrecy_of g7: Bs’ is kept secret to the server S.
- authentication_on u_s_v: The server S authenticates the user U based on V received from the message of the user U.
- authentication_on u_s_tu: The server S authenticates the user U based on Tu received from the message of the user U.
- authentication_on s_u_ts: The user U authenticates the server S based on Ts received from the message of the server S.

Box 1 **The HLPSL specification of the user.**

role user (U, S: agent, Kus, SKij: symmetric_key, Ks: public_key, H, H1, H2, H6, H7, H14, H16: hash_func, SND, RCV: channel (dy))

played_by U def=

local State: nat,

IDi, IDinew, IDj, Nj, PWi, Bi, E, Ej, Xj, V, N, N1, BBi, W, Yij, Tu, Ts, T1, T2, A, As, B, Bs, SKijNew: text, K, Q, Ay, By: message

init State := 0

transition

% Registration phase

1. State = 0/\ RCV(start) =|>

State’:= 1

%/\ Enter IDi, PWi & Bi

/\ E’ := new()/\ BBi’ := H(PWi.Bi)/\ W’ := H(H(PWi.E’).xor(h(xor(IDi,IDj)),E’))

/\ SND({IDi.W’.BBi’}_Kus)

/\ secret(E’,g1,{U})/\ secret(IDi,g2,{U,S})/\ secret(PWi,g3,{U})/\ secret(Bi,g4,{U})

2. State = 1/\ RCV({IDi.Nj.{H(Xj’).IDj.IDi.H(H(PWi.E).xor(h(xor(IDi,IDj)),E)).H(PWi.Bi)}_Xj’}_Kus) =|>

State’:= 2

/\ Ej’ := xor(E,({H(Xj).IDj.IDi.W.BBi}_Xj))

%/\ Store E, IDi, PWi & Bi in the smart card %/\ Store Ej’, IDj, & Nj in the USB

% Login and initial authentication phase

3. State = 0/\ RCV(start) =|>

State’:= 1

%/\ Insert smart card %/\ Enter PWi* & Bi*

/\ V’ := new()

%/\ Suppose b = 3

/\ N’ := H(H(H(V’)))/\ BBi’ := H(PWi.Bi)/\ W’ := H(H(PWi.E).xor(h(xor(IDi,IDj)),E))/\ Yij’ := xor(E,Ej)/\ Tu’ := new()/\ A’ := xor(xor(BBi’,W’),Tu’)/\ K’ := {IDi.IDj.Yij’.N’.A’.Tu’}_Ks

/\ SND(K’)

/\ witness(U,S,u_s_v,V’)/\ witness(U,S,u_s_tu,Tu’)

/\ secret(IDi,g2,{U,S})/\ secret(PWi,g3,{U})/\ secret(Bi,g4,{U})

4. State = 1/\ RCV(({B’.H(Yij.IDi.H(Xj)).H6(H(H(Yij.IDi.H(Xj)).Xj.As’)).H14(H(H(Yij.IDi.H(Xj)).Xj.Bs’)).Ts’}_SKij’).T1’.T2’) =|>

State’:= 2

/\ SKij’ := H(xor(N,Yij))

%/\ Confirm Ts’ %/\ Confirm B %/\ Store IDinew, ATa, ATb in the smart card %/\ Store T1, T2 in the USB

/\ request(S,U,s_u_ts,Ts’)

% Fast authentication phase

5. State = 0/\ RCV(start) =|>

State’:= 1

%/\ Enter IDinew, PWi & Bi %/\ Suppose Tlogin = 8

/\ Ay’ := H(H1(H6(H(H(Yij.IDi.H(Xj)).Xj.As)))).H(H2(H14(H(H(Yij.IDi.H(Xj)).Xj.Bs))))

/\ SND(Ay’)

6. State = 1/\ RCV({H(xor(Ay’,IDinew))}_SKij) =|>

State’:= 2

%/\ Confirm By’

end role

After executing the tool, as shown in Box 4 and Box 5 respectively, the analysis results of the proposed protocol using the OFMC and CL-AtSe backends confirm that the stated secrecy and authentication properties are satisfied for a bounded number of sessions as specified in the environment role. Thus, our scheme can resist various well-known attacks.

Box 2 **The HLPSL specification of the server.**

role server (U, S: agent, Kus, SKij: symmetric_key, Ks: public_key, H, H1, H2, H6, H7, H14, H16: hash_func, SND, RCV: channel (dy))

played_by S def=

local State: nat,

IDi, IDinew, IDj, Nj, PWi, Bi, E, Ej, X, Y, Xj, V, N, N1, BBi, W, Yij, Tu, Ts, T1, T2, A, As, B, Bs, ATa, ATb, SKijNew: text, K, Q, Ay, By: message

init State := 0

transition

% Registration phase

1. State = 0/\ RCV({IDi.H(H(PWi.E’).xor(h(xor(IDi,IDj)),E’)).H(PWi.Bi)}_Kus) =|>

State’:= 1

/\ Yij’ := {H(Xj).IDj.IDi.H(H(PWi.E’).xor(h(xor(IDi,IDj)),E’)).H(PWi.Bi)}_Xj

%/\ Store IDj

/\ SND({IDi.Nj.Yij’}_Kus)

/\ secret(Xj,g5,{S})

% Login and initial authentication phase

2. State = 0/\ RCV({IDi.IDj.Yij’.H(H(H(V’))).A’.Tu’}_Ks) =|>

State’:= 1

%/\ Confirm Tu’ %/\ Use Xj to decrypt Yij %/\ Confirm H(Xj), IDsj & IDi

/\ A’ := xor(xor(BBi.W).Tu’) %/\ Confirm A

/\ Ts’ := new()/\ B’ := xor(H(H(H(H(V’)))),Ts’)/\ IDinew’ := H(Yij.IDi.H(Xj))/\ T1’ := new()/\ T2’ := new()/\ As’ := new()/\ Bs’ := new()

%/\ Z =24, suppose T1=7, T2=10

/\ ATa’ := H6(H(IDinew’.Xj.As’))/\ ATb’ := H14(H(IDinew’.Xj.Bs’))/\ SKij’ := H(xor(H(H(H(V’))),Yij’))/\ Q’:= {B’.IDinew’.ATa’.ATb’.Ts’}_SKij’

/\ SND (Q’.T1’.T2’)

/\ witness(S,U,s_u_ts,Ts’)

/\ secret(As’,g6,{S})/\ secret(Bs’,g7,{S})

/\ request(U,S,u_s_v,V’)/\ request(U,S,u_s_tu,Tu’)

% Fast authentication phase

3. State = 0/\ RCV(H(H1(H6(H(H(Yij.IDi.H(Xj)).Xj.As’)))).H(H2(H14(H(H(Yij.IDi.H(Xj)).Xj.Bs’))))) =|>

State’:= 1

/\ X’ := H(IDinew.Xj.As)/\ Y’ := H(IDinew.Xj.Bs)/\ Ay’ := H(H7(X’).H16(Y’))

%/\ Confirm Ay

/\ By’ := {H(xor(Ay’,IDinew))}_SKij

/\ SND(By’)

end role

Box 3 **The HLPSL specification of the session role, environment role and goals.**

role session (U, S: agent, Kus, SKij: symmetric_key, Ks: public_key, H, H1, H2, H6, H7, H14, H16: hash_func) def=

local SU, RU, SS, RS: channel (dy)

composition

user (U,S,Kus,SKij,Ks,H,H1,H2,H6,H7,H14,H16,SU,RU)/\ server (U,S,Kus,SKij,Ks,H,H1,H2,H6,H7,H14,H16,SS,RS)

end role

role environment() def=

const u, s: agent,

kus, skij, kui: symmetric_key,

ks, ki: public_key,

h, h1, h2, h6, h7, h14, h16: hash_func,

u_s_v, u_s_tu, s_u_ts, g1, g2, g3, g4, g5, g6, g7: protocol_id

intruder_knowledge = {u,s,ks,ki,inv(ki)}

composition

session(u,s,kus,skij,ks,h,h1,h2,h6,h7,h14,h16)/\ session(u,i,kui,kui,ks,h,h1,h2,h6,h7,h14,h16)/\ session(i,s,kui,kui,ks,h,h1,h2,h6,h7,h14,h16)

end role

goal

secrecy_of g1, g2, g3, g4, g5, g6, g7authentication_on u_s_v, u_s_tu, s_u_ts

end goal

environment()

Box 4 **The results of the OFMC back-end.**

% OFMC

% Version of 2006/02/13

SUMMARY

SAFE

DETAILS

BOUNDED_NUMBER_OF_SESSIONS

PROTOCOL

/home/span/span/testsuite/results/WSN_e-Health.if

GOAL

as_specified

BACKEND

OFMC

COMMENTS

STATISTICS

parseTime: 0.00s

searchTime: 0.70 s

visitedNodes: 163 nodes

depth: 6 plies

Box 5 **The result of the CL-AtSe back-end.**

SUMMARY

SAFE

DETAILS

BOUNDED_NUMBER_OF_SESSIONS

TYPED_MODEL

PROTOCOL

/home/span/span/testsuite/results/WSN_e-Health.if

GOAL

As Specified

BACKEND

CL-AtSe

STATISTICS

Analysed : 2 states

Reachable : 0 states

Translation: 0.09 s

Computation: 0.00 s

## 8. Semantic Security Analysis

*Resistance to online password guessing attack*: In this case, the attacker has obtained some relevant parameters and tries to guess the password to initiate login request. Nevertheless, the server can easily observe this attack by verifying the validity of the value α of the request message k. Thus, online password guessing attack is resisted in our protocol.

*Resistance to offline password guessing attack*: The attacker attempts to collect all offline information to guess the correct password. However, the attacker does not have the private key of the server, thus he cannot decrypt message k. Similarly, the attacker does not have $sk_{ij}$, thus he is not able to decrypt message Q. Moreover, since the messages are changed in every single login, the attacker cannot use the stolen information of the previous login to compromise the current login. Besides, $PW_{i}$ is not available to the public and is computed only when the user inserts the smart card. Hence, our protocol is safe against offline password guessing attack.

*Resistance to impersonation attack*: In our protocol, the attacker cannot carry out impersonation attack without knowing password $PW_{i}$ (owing to password guessing attack resistance as stated above) and string number σ. Therefore, the attacker cannot compute the correct W and α to impersonate the user with the candidate login message. Hence, our work is free from impersonation attack.

*Resistance to replay attack*: Our protocol includes timestamp $T_{1}$ in login message $k={\left(ID_{S_{j}}||ID_{i}||y_{ij}||N||\alpha ||T_{1}\right)}^{2}\;mod\;n_{j}$; therefore, the server $S_{j}$ can easily check the validity of the message k. In addition, the user $U_{i}$ can verify the validity of the message Q by checking the timstamp $T_{2}$. Furthermore, all the messages are calculated using random number strings, which are used just once in every communication session. Thus, our protocol fully resists replay attack.

*Resistance to DoS attack*: As stated above, our protocol uses timestamp to prevent attacker from intercepting user’s message and then retransmitting it repeatedly to disrupt the server. The message $k={\left(ID_{S_{j}}||ID_{i}||y_{ij}||N||\alpha ||T_{1}\right)}^{2}\;mod\;n_{j}$ includes timestamp $T_{1}$ to prevent the attacker from retransmitting login requests to the sever. Therefore, the proposed protocol is secure from DoS attack.

*Resistance to modification attack*: This attack happens when the attacker intercepts the login message *k* and transmits a modified one to the sever. The value *k* is a ciphertext computed using public key $n_{j}$, which is only decrypted using the private key $p_{j}$ and $q_{j}$ of the server. Moreover, the attacker is still blocked by timestamp $T_{1}$ (due to the resistance to replay attack and DoS attack stated above) even when he has compromised the message k. Similarly, the message Q is protected by the session key $sk_{ij}$ and the timestamp $T_{2}$. Therefore, the proposed scheme can resist modification attack.

*Resistance to insider attack*: Since the proposed scheme does not require storage for storing the biometric data, it is not possible for a malicious legal user (attacker) to impersonate legitimate user without the correct biometric characteristic $B_{i}$. In addition, verification table is not required in our scheme. Thus, our scheme can fully prevent insider attack.

*Resistance to MITM attack*: In our protocol, the attacker cannot compromise message *k* and sends a login request to the sever since he/she is not able to compute the correct $h\left(x_{j}\right)$ for server verification without secret key $x_{j}$. Moreover, the attacker also cannot calculate the correct *k* due to the resistance to password guessing attack and impersonation attack as stated. Hence, the attacker cannot act as a middleman and our scheme is free from man-in-the-middle attack.

*Resistance to stolen smart card attack*: Suppose the smart card has been stolen and the attacker has obtained the values σ, $ID_{i}$, $PW_{i}$, and $B_{i}$. However, since the attacker does not know of the identity of the server, he cannot compute $W^{\prime }$. Besides, the attacker is unable to compute $y_{ij}$ from σ and $\varepsilon_{j}$ unless he/she steals smart card and flash drive respectively at the same time. As a result, it is not possible for the attacker to compute the correct α and k for verification. Therefore, the proposed protocol is safe against stolen smart card attack.

*Resistance to desynchronization attack*: In login and initial authentication phase, the server uses $sk_{ij}$ to encrypt acknowledgment message β and then send β to the user. The server will check the validify of the message β before accepting the session key $sk_{ij}$. Similarly, in fast authentication phase, the session key $sk_{ij}$ is accepted only when $A_{\gamma }$ and $B_{\gamma }$ have been confirmed. The user will delete the session key and restart whole process if the confirmations are not successful. Thus, desynchronization attack is resisted in our scheme.

*Provision of biometric data anonymity*: In the registration phase, biometric data $B_{i}$ and password $PW_{i}$ are computed using one-way hash function. Biometric data will not be available to public since the hash is an irreversible value. Hence, the proposed scheme provides biometric data anonymity for the user.

*Provision of forward secrecy*: The attacker attempts to use information from the past communication session to derive the key. Suppose the attacker has obtained the random strings v and b, he/she is not able to compute the session key without the values σ and $\varepsilon_{j}$ stored in the smart card and flash drive. Therefore, the proposed protocol achieves forward secrecy.

*Provision of user anonymity and untraceability*: The identity $ID_{i}$ is only included in the message $W=h(h(PW_{i}||\sigma )||\left(h\left(ID_{i}⊕ID_{S_{j}}\right)⊕\sigma \right))$. Owning to the one-way hash function, the identity $ID_{i}$ is not available to the public during communication process. In other words, the identity $ID_{i}$ is kept secret to the user $U_{i}$ and the server $S_{j}$. In addition, the attacker cannot identify any two past protocol runs initiated by the same user since the value k is computed using random number v. Therefore, the proposed scheme achieves strong user anonymity and untraceability.

Compared with previous works, Table 2 shows that our scheme is free from DoS attack, which is a vulnerability to all others. Fan and Lin [38] and Jiang et al. [39] are not secure against stolen smart card attack and desynchronization attack. Besides, Fan and Lin [38] and Zhang et al. [26] suffer from storage burden of storing biometric data in their proposed schemes. Jiang et al. [39] is not free from resist replay attack. Fan and Lin [38] is not able to resist online password guessing attack, modification attack, impersonation attack and man-in-the-middle attack. Besides, Fan and Lin [38] does not provide user untraceability. Especially, only our work provides time bound solution and fast authentication.

## 9. Performance Analysis

**Figure 7:**
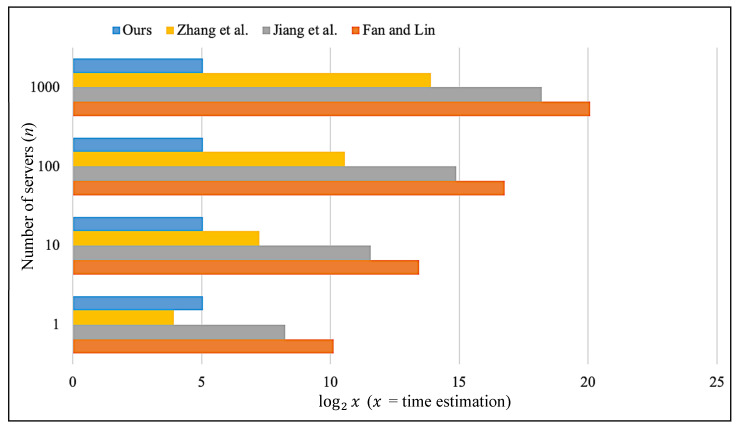
Running time of different schemes.

In this section, we provide a performance analysis to compare our scheme with its predecessor schemes. Specifically, we make a comparison with the logarithm to base 2 of the running time of each scheme. The value $\log_{2}x$ is used to compare the efficiency of the protocols where x is the rough estimation of running time (Table 3) when n (number of servers) increases from 1 to 1000. When n gradually increases, Figure 7 shows that our scheme is more efficient than the predecessor schemes. Even in single-server architecture (where n = 1), our scheme is more efficient than Fan and Lin [38] and Jiang et al [39].

## 10. Implementation of the Proposed Scheme

Consistent with the proposed system model presented in Figure 2, we present possible scenarios in a 5G-based multi-server-based healthcare system. The user can use his/her biometric sensor-enabled mobile device and body wearable sensors to obtain services from multiple servers.

- *Scenario 1*: The user can use the smart card, password, and sensor device to login to Home Care Server (*S*_1_) of Service Provider 1 to query his/her healthcare status. In addition, the user can login to healthcare data center to upload personal health information. Furthermore, the user can also login to Service Provider 2 (*S*_2_) and compute a session key to obtain remote healthcare services with caregivers.
- *Scenario 2*: With the help of continuous care across the domains, the user can login to Healthcare Service Provider 3 (*S*_3_) to upload health sensing data produced by the wearable sensors. Besides, when the user gets in community care domain, he/she can login to its healthcare server to compute session keys for using IoMT-devices through a 5G wireless network.

Furthermore, after registering with *S*_1_, *S*_2_, and *S*_3_, the user possesses (*PW_i_*, *σ_i_*, *B_i_*) and then stores them in the smart card. The public parameters (*ε*_1_, *ID_s_*_1_, *n*_1_), (*ε*_2_, *ID_s_*_2_, *n*_2_), and (*ε*_2_, *ID_s_*_2_, *n*_2_) of *S*_1_, *S*_2_, and *S*_3_, respectively, are stored in the flash drive. Consistent with user anonymity property of our proposed scheme, privacy of the user (*ID*_i_) is preserved during this communication process. Besides, using the proposed scheme, the communication between the user and the servers is safe against possible attacks specified in Section 7. For example, the attacker cannot steal the smart card (containing (*PW_i_*, *σ_i_*, *B_i_*)) and the flash drive (containing (*ε*_1_, *ID_s_*_1_, *n*_1_)) at the same time, thus the stolen smart card attack is resisted. If these three services are provided by a single healthcare institution, overhead of the proposed system is still only 33.125 ms (according to Table 3), which costs less than the methods of Zhang et al. (45.405 ms), Jiang et al. (900.42 ms), and Fan and Lin (3319.23 ms).

In addition, if service providers would like to give some discounts to specific users for their particular contribution, for instance valuable health data, the servers may use time-bound authentication solution introduced in our work for this purpose. Only authenticated users within an authorized time bound are able to get the discounts from the providers. In a hospital, time-bound authentication is also useful for physicians to set up examination schedules for specific patients.

**Figure 8:**
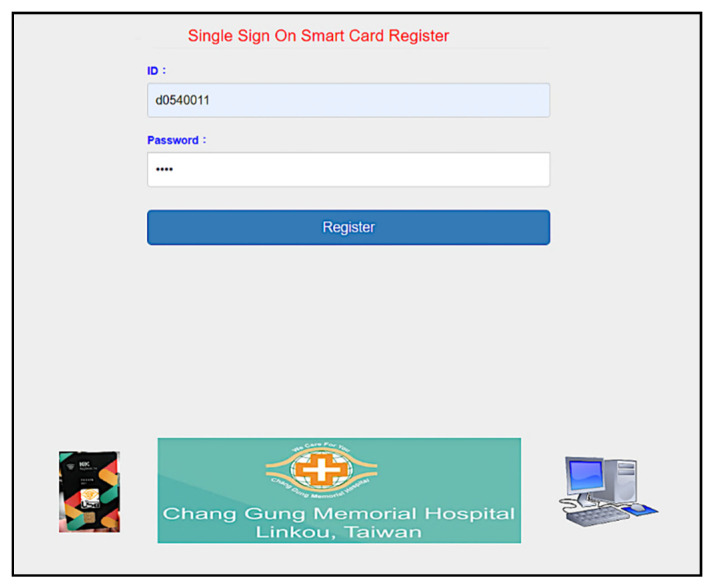
User registration.

**Figure 9:**
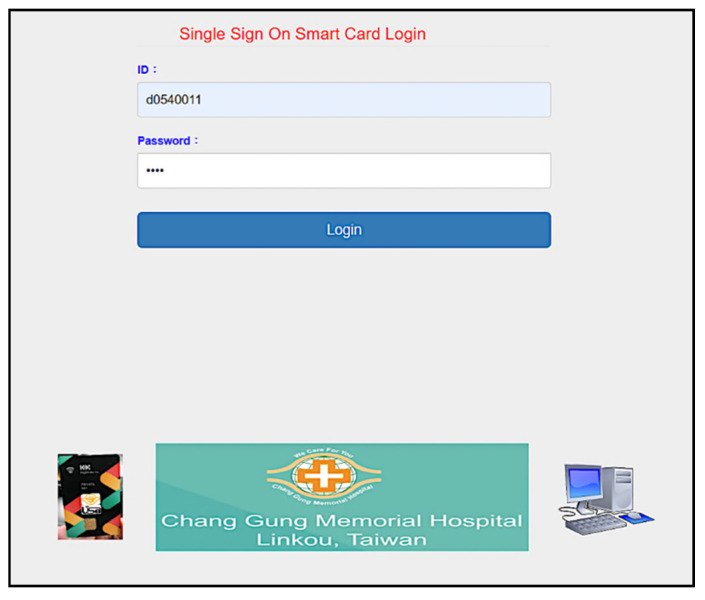
User login.

**Figure 10:**
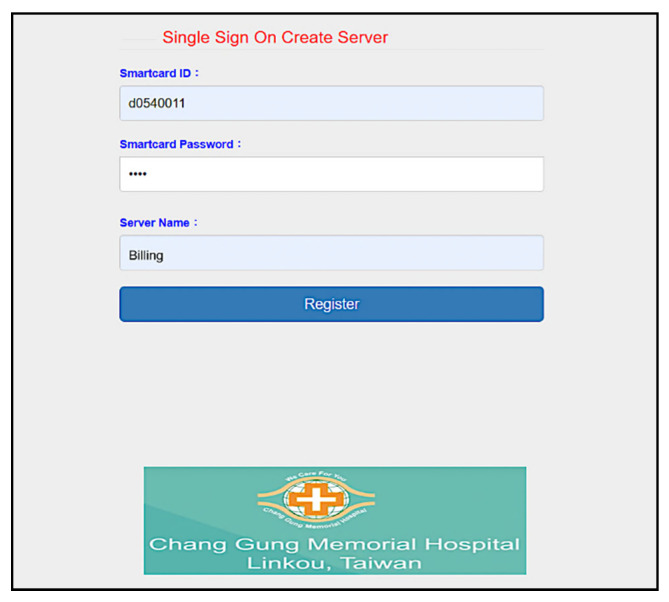
Server creation.

**Figure 11:**
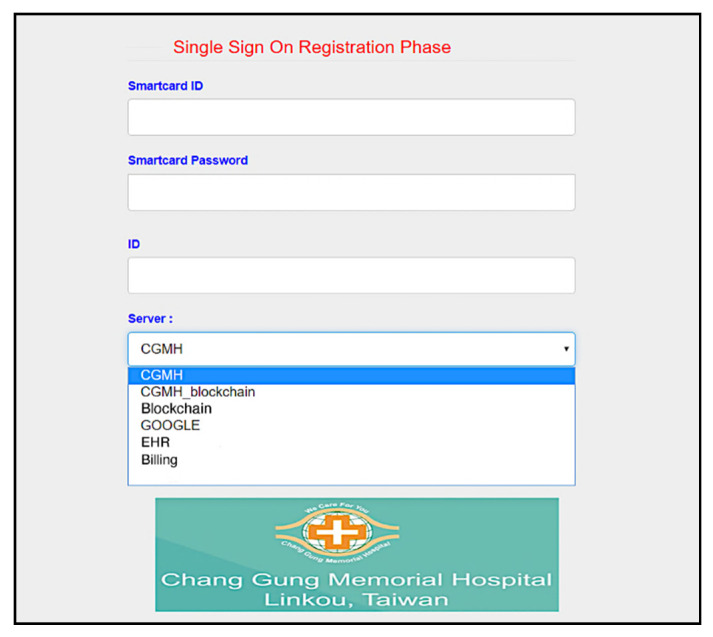
Server query.

**Figure 12:**
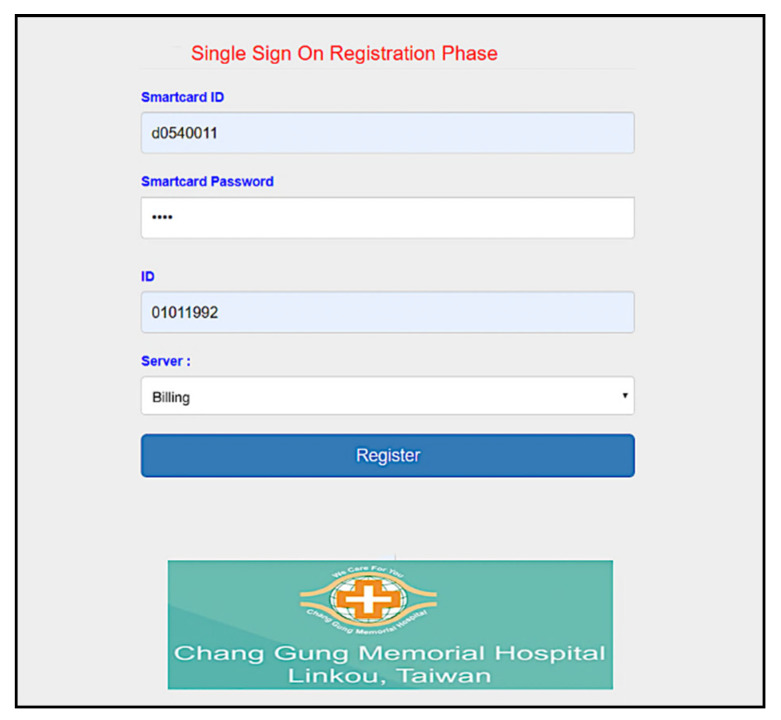
Registration with specific server.

**Figure 13:**
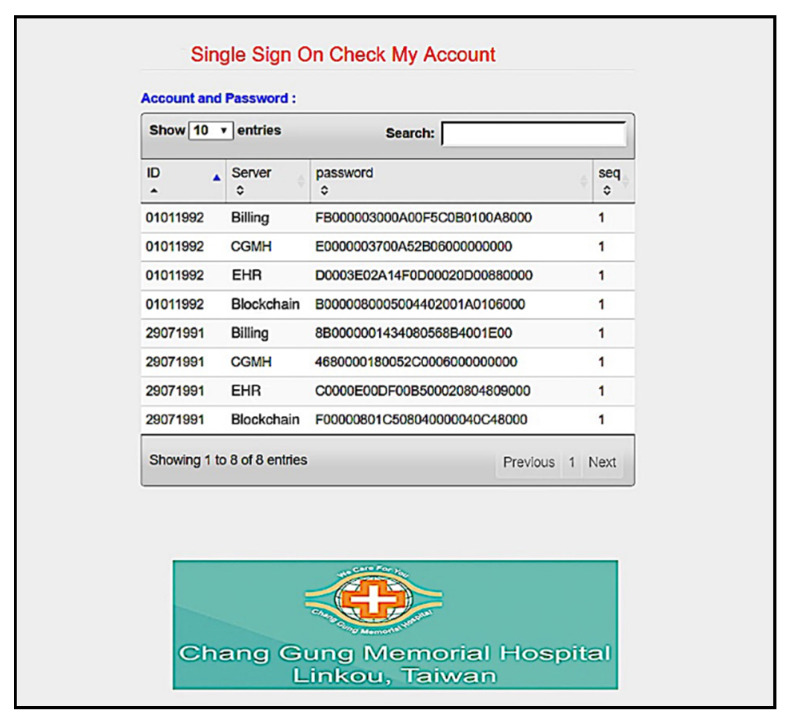
Account checking.

Furthermore, we use the Go programming language to develop a system interface, where the user uses smart card to register for using services provided by Linkou Chang Gung Memorial hospital. Multi-server architecture can be designed with single sign-on (SSO) [56]. SSO solution allows the users to access multiple applications of the same authentication provider using single identity and password. First, the user makes a registration with the server (Figure 8, and then uses registered information (including the identity *d0540011*) to login to the system (Figure 9). We allow the user to create specific servers by himself/herself in this simulation. As shown in Figure 10, the user has used the smart card to create Billing server. The user can query server information in the next step. As shown in Figure 11, several servers including *CGMH*, *CGMH_blockchain*, *Blockchain*, *GOOGLE*, *EHR,* and *Billing* have been created. Next, the user uses his/her identity, password, and an additional ID (*01011992*) to register with the desired server, for instance Billing server (Figure 12). Finally, the user can check his/her account in which specific servers (*Blockchain*, *CGMH*, *Billing,* and *EHR*) and identities (*01011992* and *29071991*) are listed with the corresponding extra passwords automatically generated by SSO-enabled system (Figure 13). By this mechanism, the user is able to obtain data from multiple services provided by the hospital.

## 11. Conclusions

The use of 5G-enabled WSN applications in IoT architecture has gained a lot of attention from the scientific community. E-health system allows e-health users to store and share their data in a more convenient way compared to the traditional healthcare system. By the support of 5G technology, healthcare data produced from sensor nodes are efficiently transited in e-health system for efficient services, better analysis reports, and faster access to treatment. In this paper, we propose a three-factor fast authentication scheme with time bound and user anonymity for multi-server e-health systems in 5B-based wireless sensor networks. Three-factor authentication scheme combining biometrics, password, and smart card ensures a high security communication for participating parties in sensor-enabled environments. User anonymity is preserved during authentication process of our protocol. Besides, the proposed protocol introduces a fast authentication for accelerating communication process. This protocol is also designed with multi-server architecture that helps save database cost and alleviate network load. In addition, time-bound authentication introduced in the proposed protocol is suitable for various scenarios in healthcare. Security proof and performance analysis results show that our work can resist more attacks and bear a rational computational cost compared to its predecessor works.

## Figures and Tables

**Table 1:** Notations used in the proposed scheme.

Symbols	Description
$S_{j}$	Server j
$U_{i}$	User i
$ID_{S_{j}}$	Identity of server j
$ID_{i}$	Identity of user i
$PW_{i}$	Password of user i
$B_{i}$	Biometric template of user i
$x_{j}$	Randomly selected string, the symmetric encryption key of the server $S_{j}$
$p_{j}$, $q_{j}$	Arbitrary big numbers, which are private keys of the server $S_{j}$
$n_{j}$	$n_{j}=p_{j}$ · $q_{j}$, the public key of the server $S_{j}$
σ, v	Randomly generated strings
b	Randomly generated value
*T*_1_, *T*_2_	Timestamp
*t*_1_, *t*_2_	Time bound
$sk_{ij}$	Session key established by the user and the server
*h*(.)	One-way hash function
⊕	Exclusive OR function
SE(), SD()	Symmetric encryption, decryption
AD()	Asymmetric decryption
${\left[\right]}_{smart\;card}$	Store information into smartcard
${\left[\right]}_{usb}$	Store information into USB

**Table 2:** Comparison of security properties.

	Fan and Lin [38]	Jiang et al. [39]	Zhang et al. [26]	Ours
Resistance to online password guessing attack	X	O	O	O
Resistance to offline password guessing attack	O	O	O	O
Resistance to impersonation attack	X	O	O	O
Resistance to replay attack	O	X	O	O
Resistance to DoS attack	X	X	X	O
Resistance to modification attack	X	O	O	O
Resistance to insider attack	O	O	O	O
Resistance to MITM attack	X	O	O	O
Resistance to stolen mart card attack	X	X	O	O
Resistance to desynchronization attack	X	X	O	O
No storage burden of biometric data	X	O	X	O
Provision of biometric data anonymity	O	O	O	O
Provision of forward secrecy	O	O	O	O
Provision of fast authentication	X	X	X	O
Provision of time-bound authentication	X	X	X	O
Provision of user anonymity	O	O	O	O
Provision of user untraceability	X	O	O	O

**Table 3:** Comparison of computational complexities.

	Fan and Lin [38]	Jiang et al. [39]	Zhang et al. [26]	Ours
Registration phase	2$T_{SED}$ + $T_{H}$ + $T_{X}$	4$T_{H}$ + 3$T_{X}$	7$T_{H}$ + 5$T_{X}$	$T_{SED}$ + 5$T_{H}$ + 3$T_{X}$
Login and authentication phase	5$T_{SED}$ + 2$T_{ASED}$ + 2$T_{H}$ + $T_{X}$	4$T_{PM}$ + 4$T_{SED}$ + 10$T_{H}$ + $T_{X}$	23$T_{H}$ + 22$T_{X}$	2$T_{SED}$ + 9$T_{H}$ + 2$T_{X}$
Password update phase	----	12$T_{H}$ + 4$T_{X}$	----	----
Total time complexities	7$T_{SED}$ + 2$T_{ASED}$ + 3$T_{H}$ + 2$T_{X}$	4$T_{PM}$ + 4$T_{SED}$ + 26$T_{H}$ + 8$T_{X}$	30$T_{H}$ + 27$T_{X}$	3$T_{SED}$ + 14$T_{H}$ + 5$T_{X}$
Total rough estimation (ms)	1106.41n	300.14n	15.135n	33.125n

n, number of servers; $T_{E}$, time for performing an exponentiation operation; $T_{PM}$, time for performing an elliptic curve point multiplication operation; $T_{SED}$, time for performing a symmetric encryption/decryption operation; $T_{ASED}$, time for performing an asymmetric encryption/decryption operation; $T_{H}$, time for performing a hash function operation; $T_{X}$, time for performing an exclusive-or operation; According to Banerjee et al. [55]: $T_{E}$ ≈ 522 ms; $T_{PM}$ ≈ 63.075 ms; $T_{SED}$ ≈ 8.7 ms; $T_{ASED}$ ≈ 522 ms; $T_{H}$ ≈ 0.5 ms; and $T_{X}$ ≈ 0.005 ms.
